# Editorial: Interactions between the mammalian main and accessory olfactory systems, volume II

**DOI:** 10.3389/fnana.2023.1343648

**Published:** 2023-12-19

**Authors:** Jorge Larriva-Sahd, Pablo Sanchez-Quinteiro

**Affiliations:** ^1^Institute of Neurobiology, Universidad Nacional Autónoma de México, Querétaro, Mexico; ^2^Department of Anatomy, Animal Production and Clinical Veterinary Sciences, Faculty of Veterinary, University of Santiago de Compostela, Lugo, Spain

**Keywords:** main olfactory system (MOS), vomeronasal system, neurochemistry, electrophysiology, morphometry, mammals

The current Research Topic, influenced by the long-lasting debate over whether the vomeronasal and main olfactory systems operate independently or in synergy, presents five distinct perspectives. These are organized into key themes: structural features, plasticity in the social-sexual brain, behavioral studies, neuropathological analysis, and a review-based article.

The research on the structural and neurochemical interaction between the olfactory and vomeronasal systems is a collaborative effort led by Pablo Sánchez-Quinteiro from the University of Santiago de Compostela, and Jorge A. Larriva-Sahd from the Universidad Nacional Autónoma de México (Ortiz-Leal et al.). This study focuses on the transition zone between the main and accessory olfactory bulbs, known as the olfactory limbus (OL), in the red fox (*Vulpes vulpes*). Moving beyond traditional laboratory rodent models, this histological and immunohistochemical investigation reveals a notably developed and complex OL in the red fox. Their findings establish the red fox as a new and significant mammalian model for exploring noncanonical olfactory pathways. This collaboration broadens our understanding of how mammals process a diverse array of chemosensory cues, offering new perspectives for future studies in neurobiology and animal behavior.

The study of the neural foundations of social bonding and sexual behavior was exemplified by the contribution by Wendy Portillo and her team from the Universidad Nacional Autónoma de México and collaborators from Emory University (Castro et al.). This research unfolds the complex ways in which social interactions and mating experiences distinctly shape neuron survival in in prairie voles. It reveals a fascinating dichotomy: while mated female prairie voles exhibit an increase in new neurons in the olfactory bulbs and the dentate gyrus of the hippocampus, males show a decrease under similar conditions. This work elegantly demonstrates how neuron survival in prairie voles is intricately influenced by their social and sexual experiences, offering a deeper understanding of the neural mechanisms behind their social bonding and monogamy.

The convergence of vomeronasal and olfactory systems in mice is addressed from a behavioral approach by the group led by Enrique Lanuza from the University of Valencia and collaborators from Jaume I and Jagiellonian universities (Pardo-Bellver et al.). Their study utilizes electrophysiological and c-Fos methods to investigate how female mice process chemosensory information in the bulbo–amygdalar network when interacting with neutral or conspecific stimuli. The research finds significant activation in the vomeronasal system, particularly with male urine stimuli, and in the cortex–amygdala transition area of the olfactory system. No significant changes were noted in the reward system and the basolateral amygdala. These results highlight the amygdala's crucial role in controlling the olfactory bulbs and integrating chemosensory information during environmental exploration in mice.

The work of Alino Martinez-Marcos and his team introduces a neuropathological perspective centered on the anterior olfactory nucleus (AON) and its involvement in Parkinson's disease (PD) (Villar-Conde et al.). Their study investigates the connection between neurodegeneration, microgliosis, and astrogliosis in the AON and the prodromal symptoms of PD, especially hyposmia. Employing stereological approaches to assess the density of neuronal and glial cells and the presence of Lewy bodies across the rostrocaudal axis of the AON, the researchers found significant differences between PD and non-PD cases. Key findings include a decrease in neuronal density in the AON in PD cases, along with an increase in Iba-1 intensity, a microglia marker, in the bulbar portion of the AON in PD patients. Notably, no changes were observed in astrocytes. The study suggests that the bulbar AON, being the most rostral part of this axis, may play a crucial role in PD pathology, potentially due to a larger area occupied by Lewy bodies in these divisions.

Finally, the review by Raúl Paredes and his team at the National Autonomous University of Mexico (Mier Quesada et al.) delves into the complex interaction between the main and accessory olfactory systems in mammals, particularly rodents. Previously thought to be separate in function and anatomy, the study reveals these systems are interconnected, processing both volatile and non-volatile odorants. This challenges earlier views on their distinct roles. Additionally, the research highlights how sexual behavior in rodents triggers neurogenesis, influencing the migration of new neurons to the olfactory bulbs. These findings suggest a larger, adaptable olfactory system at play, essential for environmental adaptation and survival, indicating a far more integrated and dynamic olfactory mechanism than previously understood.

In conclusion, this Research Topic offers an enlightening journey into the intricate world of olfactory systems, uncovering the complex interplay between the vomeronasal and main olfactory systems in mammals. The diverse studies presented here not only challenge long-standing notions about these systems operating independently but also highlight their synergistic relationship in processing chemosensory information. Collectively, these studies not only advance our knowledge in the field of neurobiology but also pave the way for future research that could reshape our understanding of how mammals interact with their environment through the sense of smell.

## Author contributions

JL-S: Conceptualization, Supervision, Writing—original draft, Writing—review & editing. PS-Q: Conceptualization, Supervision, Writing—original draft, Writing—review & editing.

